# Research on Remaining Useful Life Prediction of Control Rod Drive Mechanism Rotor Components in Floating Nuclear Reactor

**DOI:** 10.3390/s25123702

**Published:** 2025-06-13

**Authors:** Liming Zhang, Chen Wang, Ling Chen, Tian Tan, Luqi Liao

**Affiliations:** 1 School of Nuclear Science and Technology, Naval University of Engineering, Wuhan 430033, China; 2 Chongqing Pump Industry Co., Ltd., Chongqing 400030, China

**Keywords:** floating nuclear reactor, Control Rod Drive Mechanism, Remaining Useful Life prediction, Variational Mode Decomposition, deep learning

## Abstract

Aiming at the difficult problem of predicting the running state of the rotor of a Control Rod Drive Mechanism (CRDM) in a floating nuclear reactor, this paper proposes a Remaining Useful Life (RUL) prediction method based on Variational Mode Decomposition and Bidirectional Long Short-Term Memory (VMD-BiLSTM). Firstly, a bench experiment of the CRDM is carried out to collect the full operational cycle (full-stroke) vibration signals of the CRDM. Secondly, the collected data are decomposed based on the VMD, and the typical vibration signals at different stages of the experiment are used to verify this method and comprehensively mine the degradation characteristics. At the same time, the time-frequency domain feature analysis is carried out on the original vibration data, and the changing trends of the extracted features are carefully analyzed. Five feature quantities closely related to the degradation trend of the rotor of the CRDM are screened out, and the corresponding health indicators are constructed in combination with the stroke. Finally, the life prediction of the rotor of the CRDM is realized through the BiLSTM method. Then, the comparison experiments with other methods are carried out, and the experimental results show that the method proposed in this paper has high accuracy and reliability and can effectively solve the RUL prediction problem of CRDM, which provides a strong support to ensure the safe and stable operation of floating nuclear reactors.

## 1. Introduction

The Control Rod Drive Mechanism (CRDM) is a servo mechanism of the control system and safety protection system of a floating nuclear reactor, and it is the only device with relative motion inside the reactor [1]. The specific function of the CRDM is to drive the control rod assembly to move continuously up and down through the sliding friction motion between the rollers and the screw rod, so as to complete the start-up, power regulation, and shutdown of the reactor. It is one of the key devices that directly affect the normal operation, safety, and reliability of a nuclear reactor. When the CRDM is in operation, the wear between the rotor and the screw rod will cause the split rotor to be unable to hold the screw rod tightly, and then the control rod cannot be lifted normally or may even drop. Once the CRDM malfunctions or reaches the end of its service life, it will have a great impact on the normal operation of the nuclear power plant [2]. Therefore, it is of vital importance to conduct predictive research on the RUL of the rotor components of the CRDM in a floating nuclear reactor.

The research methods of RUL prediction can mainly be divided into the remaining life prediction method based on physical models [3,4] and the remaining life prediction method based on data-driven models [5,6]. The prediction method based on physical models uses physical or mathematical models for life prediction. The models have the characteristic of degradation, and specific models are required for specific devices. Wang et al. established an empirical physical model for mechanical equipment based on the particle filter (PF) model to conduct research on the remaining useful life [7]. Liao et al. proposed a crack growth rate model under multi-axial loading to explain the short fatigue cracking behavior [8]. Although physical models can accurately reflect the degradation process of rotating machinery, due to the different degradation processes of various rotating machinery, the model establishment is complex and the actual operating environment of the drive mechanism may not be taken into account during the modeling process [9]. Therefore, the remaining life prediction method based on physical models has its inherent defects. The data-driven method has been a hot topic in the research direction of life prediction in recent years [10,11]. This method is specifically divided into remaining life prediction based on empirical models and remaining life prediction based on intelligent models. The remaining life prediction based on empirical models mostly adopts traditional methods. Ren et al. synchronously predicted the RUL of multiple bearings through a method combining the time-frequency domain and achieved good prediction results [12]. Matias et al. proposed an improved mean multi-scale fuzzy entropy (IMMFE) that combines complementary ensemble empirical decomposition with adaptive noise, and extracted the characteristic values of classic rolling bearings as new performance degradation evaluation indicators to improve the correlation of residual life characteristics [13].

With the development of big data and computer technology, many deep learning methods have also been applied to the life prediction of various types of rotating machinery [14,15]. Wan et al. studied the remaining useful life of bearings by using a deep learning method that integrates convolutional neural networks and long short-term memory neural networks [16]. Yu et al. predicted the remaining useful life of data with a non-full life cycle through a bidirectional long short-term memory neural network model [17]. Li et al. proposed a new data-driven prediction method using a deep convolutional neural network, which achieved high precision in RUL prediction [18]. Mao et al. proposed a novel selective transfer learning method for cross-machine RUL prediction [19]. Liu et al. proposed a novel Deep Reinforcement Learning (DRL)-based prediction method that integrates a denoising autoencoder and a converter architecture to build a robust DRL policy network capable of extracting high-quality features from X-ray records to capture subtle damage progression in material structures [20]. This data-driven prediction method gets rid of the limitations of the original manual experience and model establishment and has a wide range of applications and good application prospects [21,22].

In the research field of the CRDM, Zhu et al. proposed a method that uses a dynamic Bayesian network to characterize the correlations among various components of a multi-stage task system and between different stages. While quantifying the performance-degrading components using a hidden Markov model, they achieved the synchronous update of the components’ states and degradation rates based on the observed data [23]. Wang et al. proposed a dynamic time convolution network (DTCN) based on dynamic activation function and attention mechanism for the remaining useful life prediction of a CRDM [24]. Liu et al. proposed a CRDM vibration signal denoising method based on fully integrated empirical mode decomposition with adaptive amplitude correction noise and spectral kurtosis, which can deeply reduce the vibration signal of CRDM [25]. Jiao et al., aiming at the non-stationary and strongly noisily distorted signals existing in the collected vibration signals of the CRDM, proposed a CRDM roller state evaluation method based on an evaluation function and an error backpropagation network [26]. Xu et al. proposed a high-precision CRDM fault detection and diagnosis method combining a grid cycle unit autoencoder (GRU-AE) and random forest (RF) [27]. Li et al., in response to the problem of how to select health indicators in the prediction of the remaining useful life of the roller-screw pair of a reactor’s CRDM, adopted the negative log-likelihood probability index based on the generative topology mapping algorithm as the health status index of the roller-screw pair and proposed a new life prediction model [28]. Regarding the current research status of the CRDM, the existing research faced the following problems: the CRDM operates in a high-temperature and high-pressure coolant, and its working environment is inconvenient to simulate. In actual operation, a fixed rod speed will not be adopted, but it will change according to the actual operating conditions and requirements, and its degradation trend may be different from that under laboratory conditions.

Based on the above research status of the life prediction of a CRDM, current research on life prediction is not accurate enough for the prediction of the drive mechanism and the fitting degree of the results after neural network learning is not high [29]. The fault samples used in some studies were simulated under laboratory conditions, and there may be differences in the operating status of the equipment, so the results of the neural network algorithm learning may not be satisfactory. At the same time, as an electromechanical hybrid device, the CRDM operates in a high-temperature and high-pressure coolant. It is affected by the complex mechanical structure and harsh working environment and will be influenced by the noises of various pieces of mechanical equipment. Most of the existing studies used methods based on Support Vector Machines (SVM), Convolutional Neural Networks (CNN), etc. [30,31]. Currently, compared with traditional methods, the Long Short-Term Memory (LSTM) neural network method in machine learning can better extract the operating status of the equipment.

Aiming at the deficiencies of the existing work, this work conducted a study on the prediction of the RUL of a CRDM based on VMD-BiLSTM: Firstly, the full-life vibration signals of the CRDM collected in the bench experiment of the control rod drive mechanism were utilized, the collected vibration signals were preprocessed, and the abnormal data were removed. Secondly, in view of the modulation phenomenon of the vibration signals that occur during the operation of a CRDM, Variational Mode Decomposition (VMD) was adopted for the demodulation processing. Then, based on the analysis results of the vibration signal demodulation, the modal components related to the degradation state of the rollers were extracted. Time and frequency domain analysis methods were used to obtain the characteristic quantities that can directly represent the wear state of the CRDM rotor. According to the degradation trend of the rotor, the early fault samples with a high degree of consistency with the actual operation of the equipment were extracted. Finally, a life prediction model for the RUL of the CRDM was constructed based on this method, and the Long Short-Term Memory (LSTM) neural network method was used for multiple iterations to improve the prediction accuracy of the model.

The rest of this article is summarized as follows. Section 2 introduces the theoretical methods of VMD and BiLSTM. Section 3 introduces the CRDM accelerated life test platform in detail. The proposed method was used to conduct a series of experiments on the CRDM full-life vibration data set and compared with other methods. Finally, the conclusion is given in Section 4.

## 2. Theoretical Methods

### 2.1. Variational Mode Decomposition

VMD can decompose the original signal into a finite number of bandwidth modal components that possess certain characteristics of the original signal. This method is a completely non-recursive signal decomposition approach, which can effectively solve problems such as mode mixing and end effects and improve the accuracy and stability of signal decomposition [32]. The following are the basic steps of VMD decomposition:

(1) Construct the variational features of the signal. The significance of this step mainly lies in minimizing the sum of the estimated bandwidths of the intrinsic mode components. The constraint condition of this step is specifically manifested as the sum of all intrinsic mode components should be equal to the original signal. The specific expression of this step is as follows:

(1) $$\min\left\{u_{k}\right\}\left\{\omega_{k}\right\}\left\{\sum_{k=1}^{K}{\left\|\partial_{t}\left[\left(\delta (t)+\frac{j}{\pi t}\right)\cdot u_{k}(t)\right]\right\|}_{2}^{2}\right\}$$

(2) $$s.t.\;\sum_{k=1}^{K}u_{k}(t)=f(t)$$

In the formula, $u_{k}$ is the intrinsic mode component; *k* is the center frequency of $u_{k}$; *K* is the number of decompositions; $u_{k}(t)$ is the Dirac function; and f(t) is the original signal.

(2) Transform the variational features of the signal. In this step, the penalty factor and the relevant multiplier mentioned previously are adopted to achieve the conversion between the constrained and unconstrained variational problems. This conversion aims to ensure the accuracy of signal reconstruction while maintaining the stringency of the constraint conditions. The transformed form is as follows:

(3) $$\begin{array}{ll} L\left(\left\{u_{k}\right\},\left\{\omega_{k}\right\},\lambda \right)= & \alpha \sum_{k}{\left\|\partial_{t}\left[\left(\delta (t)+\frac{j}{\pi t}\right)\cdot u_{k}(t)\right]\right\|}_{2}^{2}\\ & +{\left\|f(t)-\sum_{k}u_{k}(t)\right\|}_{2}^{2}+\lambda (t),f(t)-\sum_{k}u_{k}(t) \end{array}$$

(3) Solve the variational features. Use the alternating direction method of multipliers to solve the unconstrained problem in the formula.

Iteratively obtain $u_{k}$ and $\omega_{k}$ through the following equations.

(4) $${\hat{u}}_{k}^{n+1}(\omega )=\frac{\hat{f}(\omega )-\sum_{i\neq k}{\hat{u}}_{i}(\omega )+\frac{\hat{\lambda }(\omega )}{2}}{1+2\alpha {\left(\omega -\omega_{k}\right)}^{2}}$$

(5) $$\omega_{k}^{n+1}=\frac{\int_{0}^{\infty }\omega {\left|{\hat{u}}_{k}^{n+1}(\omega )\right|}^{2}d\omega }{\int_{0}^{\infty }{\left|{\hat{u}}_{k}^{n+1}(\omega )\right|}^{2}d\omega }$$

In the formula, $\hat{f}(\omega )$, ${\hat{u}}_{i}$(*w*), and ${\hat{u}}_{k}$ are the Fourier transforms of *f*(*t*) and *u*(*t*), respectively.

(4) Judgment of the stopping condition. The essence of this algorithm is a process of searching for the optimal solution during the iterative process. It is necessary to set an appropriate stopping condition to ensure that the algorithm converges to a stable solution. For example, the center frequency and the modal components no longer change significantly, that is, the result is a convergent process. When one of these conditions is met, the iterative process terminates and the final modal components are output.

(5) Post-processing of modal components. After the VMD decomposition, the original signal is decomposed into a series of intrinsic modal components with finite bandwidths. These components may still require further processing, such as filtering, noise reduction, etc., to improve the quality and interpretability of the signal. In addition, according to the actual application requirements, operations such as feature extraction, classification, or identification can be performed on the modal components.

### 2.2. Bidirectional Long Short-Term Memory

The LSTM network is a variant of the recurrent neural network. Its core mechanism lies in the use of a series of carefully designed related units to achieve fine control over the input and output processes of processed data and labels. The related units mainly include the forget gate, input gate, and output gate. By screening and controlling the input data, they can effectively retain and update the information of the cell state. Through the precise screening and effective control of the input data, the effective retention and timely update of the cell state information are achieved. At the specific execution level, the LSTM network adopts a multi-time and step-by-step strategy. In each time period, the network receives relevant data and relevant states and conducts relevant processing according to the relevant states of the previous time period and the current input data to obtain the relevant states of one time period and another actual time period. Through continuous and repeated operations, the LSTM network is able to process time series information and continuously update and learn from it [33,34].

(6) $$f_{t}=\sigma \left(W_{f}h_{t-1}+U_{f}x_{t}+b_{f}\right)$$

(7) $$i_{t}=\sigma \left(W_{i}h_{t-1}+U_{i}x_{t}+b_{i}\right)$$

(8) $$o_{t}=\sigma \left(W_{o}h_{t-1}+U_{o}x_{t}+b_{o}\right)$$

(9) $${\tilde{C}}_{t}=\tanh\left(W_{c}h_{t-1}+U_{c}x_{t}+b_{c}\right)$$

(10) $$C_{t}=f_{t}*C_{t-1}+i_{t}*{\tilde{C}}_{t}$$

(11) $$h_{t}=f_{t}*\tanh\left(C_{t}\right)$$

Activation function: An activation function is used in this structure, which can preprocess relevant information and is helpful for the further processing of input data, as shown in Equation (6).

Forget gate: It can control the deletion of data. The main function of the forget gate is to determine the unnecessary information in the memory cell. The forget gate generates a normalized matrix based on the states of the current time and the previous time, indicating the range of data that must be deleted in the obtained normalized matrix. The higher the priority of the data to be deleted, the greater the probability that the corresponding data will be deleted from the corresponding cell, as shown in Equation (8).

Input gate: It controls the entry of data into the network. The role of the input gate is to decide which information needs to be input into the memory cell. The input gate will generate normalized new data according to the input in the forward time period and the information input in the backward time period, representing the relevant weights of the input in each dimension. When the input weight is larger, the greater is the degree to which the data are input into the corresponding cell, as shown in Equations (9)–(11).

Output gate: It sends out the data that have entered the relevant cell. The role of the output gate is to decide which information needs to be sent to the next cell. The output gate will generate normalized new data according to the input in the forward time period and the information input in the backward time period, representing the relevant weights of the output in each dimension. When the output weight is larger, the greater is the degree to which the data are output to the corresponding cell.

Although LSTM performs excellently in processing long sequence data, like the Recurrent Neural Network (RNN), it can still only process data unidirectionally, relying on the data from the past moments to predict the next data while ignoring the data information from the future moments. In order to further excavate the hidden information in the time series, we can add a delay between the input and output of the neural network, incorporating the information from the future moments into the time step of the current network for predicting the data at future moments. However, the more future data are added, the more likely it is the prediction effect of the network will decline.

To solve this problem, we used the BiLSTM neural network. The BiLSTM is composed of a forward LSTM and a backward LSTM. The forward LSTM trains the time series data along the forward direction, while the backward LSTM trains the time series data along the reverse direction. Both of these two layers of LSTM are connected to the relevant structures in the network, enabling the neural network to use all the data for training during the training process. In this way, while improving the accuracy of the model, the BiLSTM also makes better use of the information in the time series data.

The BiLSTM consists of a forward LSTM layer and a backward LSTM layer, each processing the input sequence in opposite directions (past to future and future to past). Both layers share the same input data but learn independent hidden states. The outputs of the two layers are concatenated and fed into a fully connected layer for regression. During training, the network optimizes weights using backpropagation through time (BPTT), minimizing the mean squared error between predicted and actual RUL values. This clarifies the training process and network structure. The output of the forward LSTM layer for the input sample, together with the output of the backward LSTM layer, jointly determines the value passed into the hidden layer to obtain the output of the BiLSTM. Its update formulas are as follows:

(12) $$\vec{h_{t}}=\mathrm{LSTM}\left(x_{t},\vec{h_{t-1}}\right)$$

(13) $$\overset{\leftarrow }{h_{t}}=\mathrm{LSTM}\left(x_{t},\overset{\leftarrow }{h_{t-1}}\right)$$

(14) $$y_{t}=\bar{W}\vec{h_{t}}+\bar{W}\overset{\leftarrow }{h_{t}}+b_{y}$$

where $\vec{h_{t}}$ is the weight matrix from the forward LSTM layer to the output layer, $\overset{\leftarrow }{h_{t}}$ is the weight matrix from the backward LSTM layer to the output layer, and $b_{y}$ is the bias matrix of the output layer.

### 2.3. The VMD-BiLSTM Method

**Figure 1:**
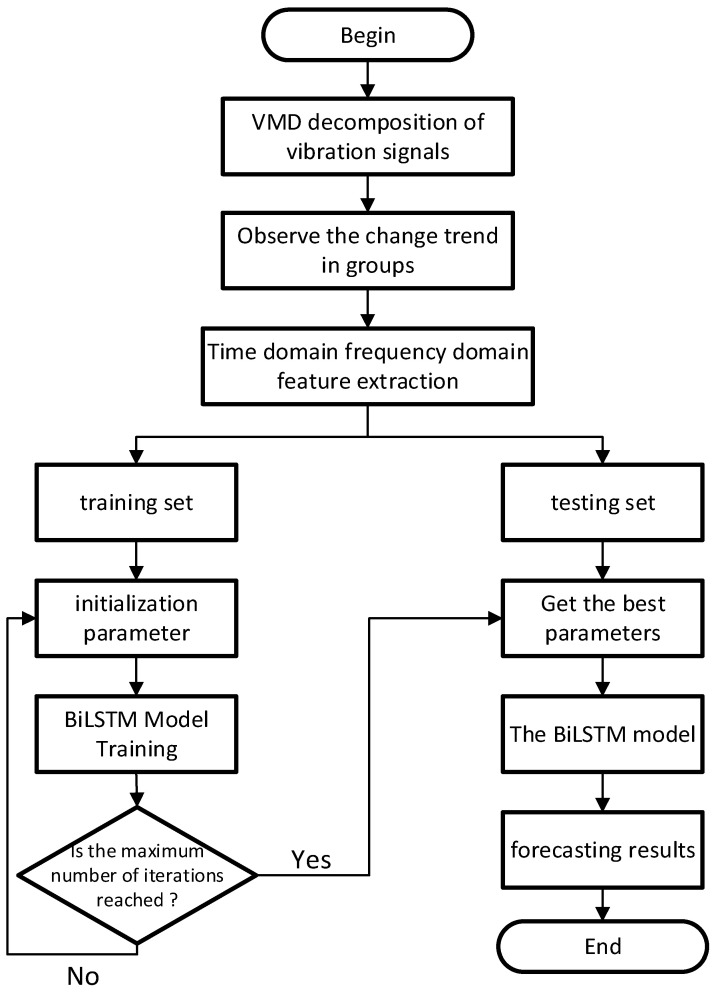
The prediction process of the RUL of the CRDM based on VMD-BiLSTM.

While effectively utilizing the sequential information of the data, the BiLSTM network demonstrates excellent retention capabilities for achieving the efficient prediction of time series data as well as the ability to select between short-term and long-term data. In addition, during the training process, the neural network can implicitly extract features from the data, thus avoiding the cumbersome feature extraction process in traditional methods that relies on empirical knowledge. In view of this, based on VMD and BiLSTM, this study proposed a method for predicting the remaining useful life of the rotor. The specific method process is shown in Figure 1.

The detailed steps are as follows:

(1) In the signal acquisition stage, comprehensively collect the vibration signal data of the rotor throughout its life cycle, determine the rotor’s degradation trend as the standard for data classification, and use the real degradation features of the rotor as labels to be imported into the network for training.

(2) In the signal analysis stage, perform short-time Fourier transform and Hilbert transform on the collected original vibration signals, conduct signal demodulation and envelope spectrum analysis, extract the signals, and perform normalization processing to ensure better prediction results after model training.

(3) In the data set division stage, scientifically and reasonably divide the preprocessed data into a training set and a test set to ensure the independence of the model’s training and testing processes. By using the preprocessed data and the test set, we effectively avoid the occurrence of over-fitting or under-fitting phenomena.

(4) In the neural network training stage, use Variational Mode Decomposition (VMD) to decompose and train the envelope signals of the bearing degradation data in the set. Import the data into the BiLSTM neural network for deep learning to train the potential temporal relationships between the rotor degradation data, further discover the data features, and output the predicted values.

(5) In the model testing stage, for a well-trained and comprehensively and accurately evaluated model, use an independent test set and objective and quantifiable evaluation indicators to test the prediction effect.

## 3. Experimental Research

### 3.1. CRDM Life Test

#### 3.1.1. CRDM Monitoring Platform

The data used in this study were derived from the full-life vibration signals of the CRDM collected during the CRDM bench test. The following is the real-time monitoring scheme for the RUL of the CRDM on the bench:

Currently, there are many methods for equipment state monitoring commonly used in floating nuclear power plants. An appropriate method needs to be selected according to the monitoring object. We can measure the acceleration signals transmitted from the CRDM to the outer shell. Its advantages include mature extraction and analysis methods, high signal sensitivity, and easy judgment of the corresponding RUL of rotating machinery. When the CRDM, the research object of this paper, is in the working state, the meshing operation of its rollers and the screw rod will generate relatively obvious vibration signals. By analyzing the changes in the vibration signals, the health state of the rotor in the RUL can be fully reflected. Therefore, this study used the vibration monitoring method to monitor the state of the CRDM. Since the rotor of the CRDM works within the pressure-bearing boundary of the primary circuit, the high-temperature and high-pressure environment makes it difficult to directly monitor its state. We adopted a non-contact detection method to collect the RUL data of the CRDM.

**Figure 2:**
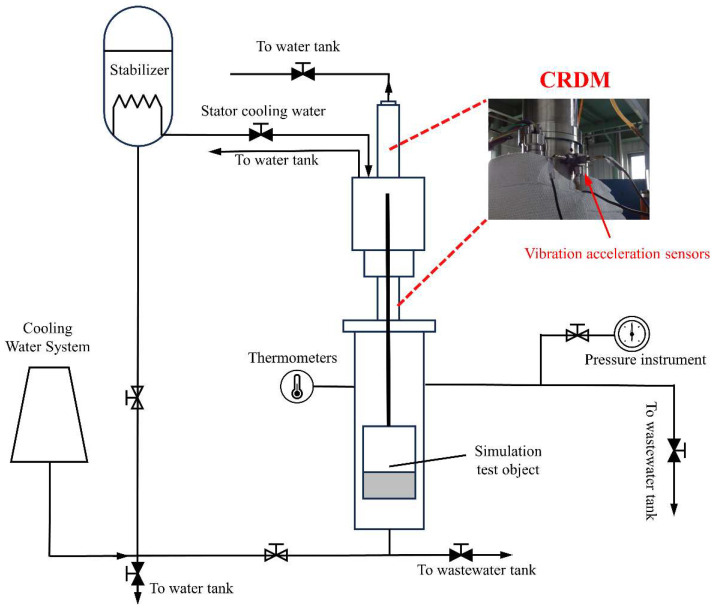
Schematic diagram of CRDM life test platform.

In a typical CRDM life test (as shown in Figure 2), the CRDM is powered and the power-supply phase sequence is adjusted to make the rotor drive the screw rod to reciprocate between the upper and lower limits, simulating the actual operation process of the equipment. Because the reactor is required to operate in a high-temperature and high-pressure environment, this environment helps to maintain the stability and safety of the reactor and also helps to prevent the leakage of radioactive materials. In order to simulate the actual operating environment of a CRDM, we conducted a thermal test, which simulated the basic situation of the driving mechanism of the actual equipment during operation. This work mainly monitored the thermal state of the driving mechanism and studied the vibration characteristics of the rotor during actual operation through state monitoring, so as to make a useful exploration for life prediction on actual equipment.

**Figure 3:**
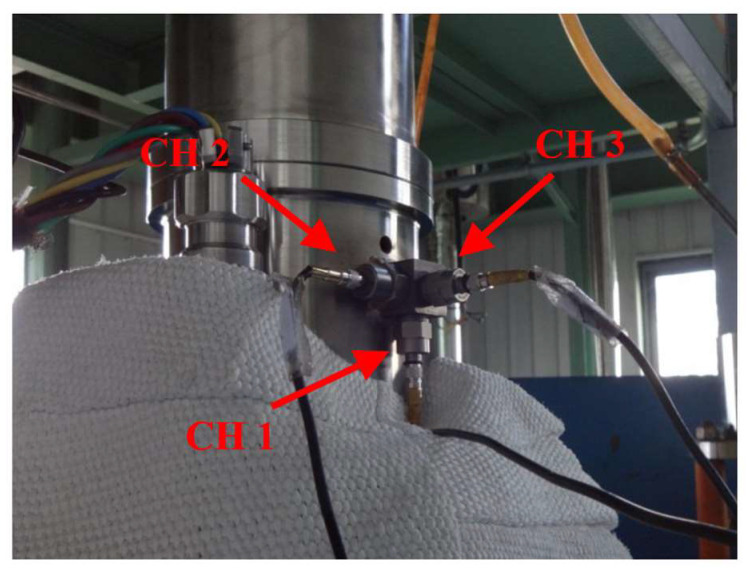
Arrangement of the acceleration sensors.

According to the structure of the CRDM, the sensor should be installed at the position closest to the rotor and with the least number of state signal transmission surfaces. Therefore, the measurement point was selected to be arranged on the adapter block welded outside the compression nut. Considering the characteristic that the vibration signal has multiple transmission surfaces, in order to monitor the vibration signals of the rotor in the axial, radial, and tangential directions, three acceleration sensors were simultaneously arranged on the adapter block (as shown in Figure 3) for real-time monitoring. The original data collected by the three sensors were marked as CH1, CH2, and CH3, respectively, and the sampling rate was set at 5000 Hz.

**Figure 4:**
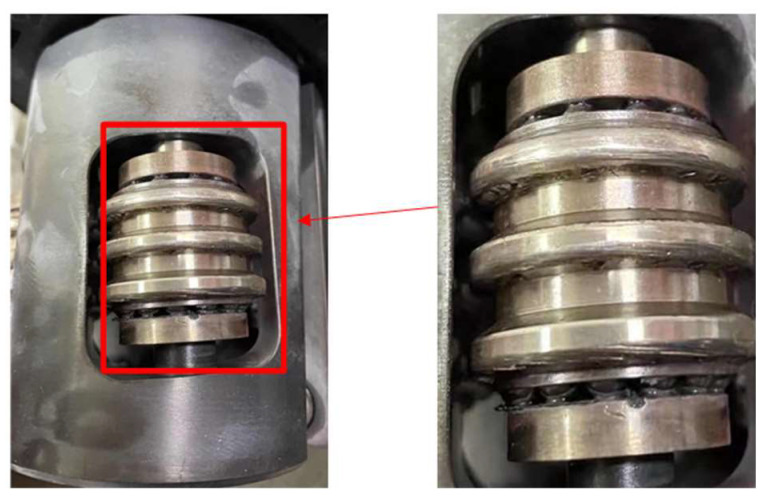
The degradation phenomenon of the roller.

At the same time, during the operation of the CRDM, the energization of the split rotor components forced the lower part of the rotor to attract. The four rollers, which were metal components, clamped the screw rod to form a basic transmission mechanism. Thus, the rotation of the rotor drive the movement of the screw rod. The friction between the rollers and the screw rod lead to a mechanical degradation process. Losses such as wear or pits appeared on the surface of the roller components (as shown in Figure 4), and the vibration signals changed.

After completing the signal acquisition work on the above-mentioned acquisition system, subsequent analysis and comparison were carried out on the collected original vibration signals. Firstly, the original vibration data were converted into a one-dimensional matrix array of the double type. Then, the signal images of the acceleration data of each channel collected in the time domain were drawn to facilitate the comparison and analysis.

**Figure 5:**
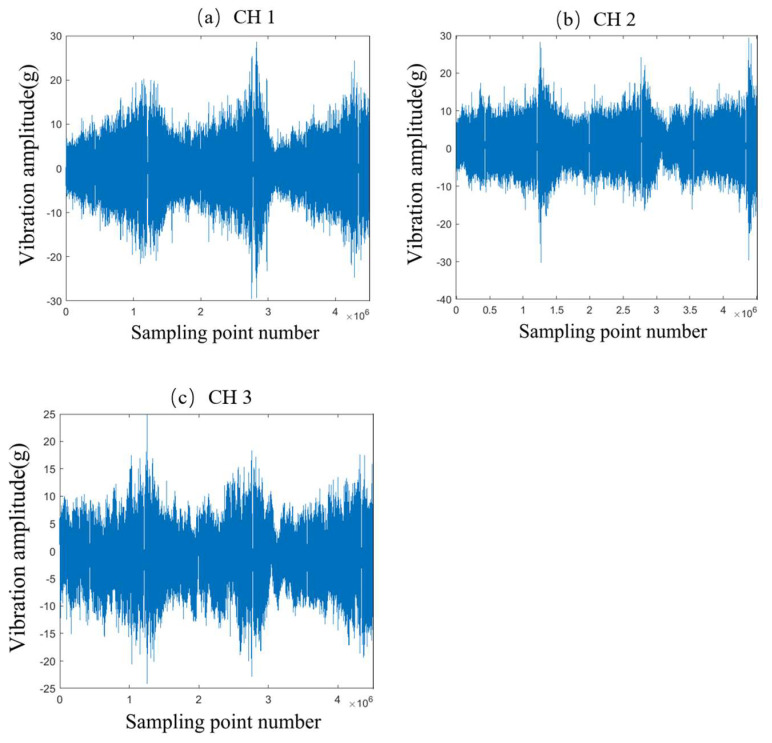
Original vibration signals in the early stage of the test.

As shown in Figure 5, the data of the three channels were the results of the noise generated by the collision and friction of the internal components of the rotor parts, which was transmitted to the outer shell and then read into the rotor condition monitoring system by the piezoelectric acceleration sensor. The first image in this figure is the axial channel, that is, the image of the acceleration vibration signal collected by the CH1 channel, which was perpendicular to the ground plane during installation. Compared with the waveforms of the other two images, the extra vibrations in the image collected by this channel were obviously smaller. Combining the installation position of the sensor and the actual situation of the internal components of the CRDM, it was known that, for the channel parallel to the axis of the rotor, the influence of the precession of the remaining components in the drive mechanism received by it was relatively small, and the other complex noises received by it were less, which provided a great deal of help for the signal processing work carried out in subsequent research. Therefore, this work used the data of sensor 1 for subsequent experiments.

#### 3.1.2. Preprocessing of Vibration Data During the Rotor Degradation Process

During the signal acquisition process, we selected 5000 Hz as the sampling frequency of the piezoelectric accelerometer. Since the CRDM is a low-speed rotating device and its degradation process is a long-term process, there is not a large amount of high-frequency noise inherently. Therefore, a sampling rate of 5000 Hz was sufficient. The operation of the CRDM is mainly divided into several working conditions, such as lifting up, inserting down, and dropping the rod. During the actual operation of the equipment, unnecessary noise will be generated when the control rod reaches the upper and lower limits. Especially for the axial signal, the vibrations when reaching the top and the bottom will be represented by obvious impact signals. These additional vibration signals cannot reflect the operation status of the rotor of the CRDM. Instead, they may be misjudged as the degradation characteristics or abnormalities of the control rod failure.

**Figure 6:**
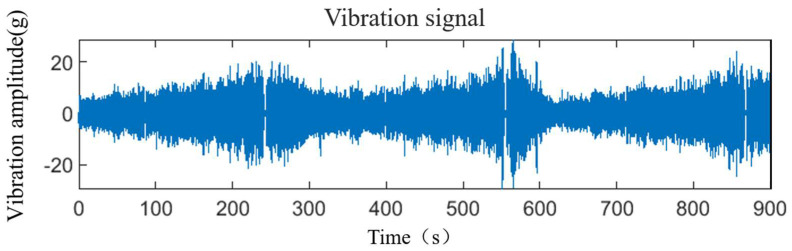
The preprocessed signal.

Therefore, in the research process for this paper, we used the experience to manually separate the unwanted part of the signal, only retained the vibration signal when the control rod was raised and inserted, and directly deleted the obvious impact signal. The remaining signals were processed by sliding sampling. The final processed vibration signals were from different strokes of the control rod drive mechanism. The preprocessed vibration signals were 900 s as a group (as shown in Figure 6), a total of 125 groups for subsequent analysis.

### 3.2. Analysis of Degradation Characteristics of the CRDM

Based on the vibration signals after demodulation processing, combined with the results of characteristic frequency analysis, we conducted testing and analysis after demodulation processing (such as time-domain characteristic values like mean value, standard deviation, root mean square amplitude, RMS (root mean square), peak-to-peak value, skewness, etc., and frequency-domain characteristic values such as mean frequency, standard deviation, root mean square frequency, etc.) of the commonly used characteristic parameters for faults of rotating machinery. We found the characteristic values that can directly represent the degradation trend of the CRDM, providing characteristic data support for the operation status prediction work of the CRDM rotor components in the subsequent time.

#### 3.2.1. Analysis Results of the Vibration Signal by VMD and EMD

**Figure 7:**
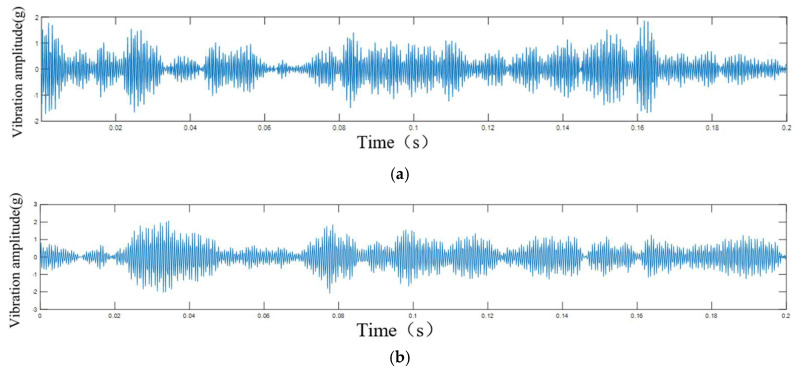

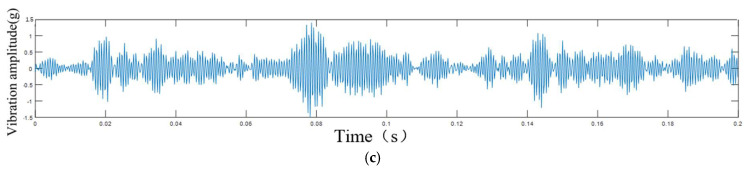
The analysis results of the first three layers of VMD: (**a**) IMF1; (**b**) IMF2; (**c**) IMF3.

**Figure 8:**
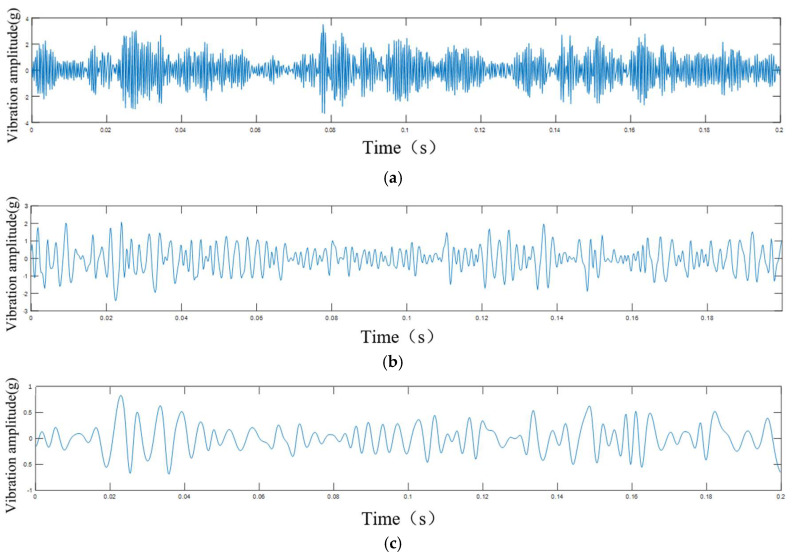
The analysis results of the first three layers of EMD: (**a**) IMF1; (**b**) IMF2; (**c**) IMF3.

According to the bench test of the CRDM, by comparing the collected data with the actual travel schedule, we divided the operation process of the rotor components of the CRDM into the initial degradation stage, the intermediate degradation stage, and the stage on the verge of failure. For the vibration data throughout the entire lifespan of the rotor of the CRDM, we decomposed it into ten IMF components using EMD and VMD, respectively, and showed the results of the first three layers of decomposition (shown in Figure 7 and Figure 8). The final signal processing method used was judged by comparing the two methods.

By observing the first three components after EMD decomposition, it was found that most of the energy of the data after EMD decomposition was retained in the IMF1 component, the other two components contained less energy, and the first component still retained the modulation characteristics of the signal of different frequencies (shown in Figure 8), which was unfavorable for the prediction of the next input model.

After observing the first three components after the VMD decomposition, it was found that the frequency features were retained in IMF1 to IMF3 after the decomposition, the periodic features of such rotating machinery as the rotor of the control rod drive mechanism were obviously retained in the three components (shown in Figure 7), and the signal modulation phenomenon was weaker than that of the original data and the EMD decomposition data; therefore, for this paper, we chose the VMD variable modal decomposition. Modal decomposition for the acquisition of the original vibration signal processing, the use of VMD to decompose the signal into ten IMF components, and the first three components were summed.

In order to verify the effect of VMD decomposition, and at the same time to judge the initial rotor degradation trend, the following three stages of degradation start, mid-degradation, and imminent failure of the IMF1 components of the time domain, frequency domain, and envelope were analyzed to analyze the characteristic frequency of each component of the rotor of the control rod drive mechanism and to determine the results of the filtering.

For this paper, the fast Fourier transform was used to demodulate the IMF1 components at the three stages in order to convert the time-domain signals into frequency-domain signals; then, the Hilbert transform was used to obtain the envelopes of the corresponding signals; and the rotor degradation status was judged by the spectrograms and the envelope diagrams.

**Figure 9:**
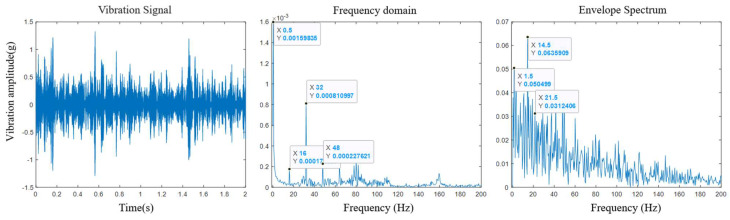
Demodulation results in the early stage of the experiment.

Through data mediation and analysis, the analysis results of the rotor components in the initial stage of the test are shown in Figure 9, which clearly presents the frequency domain signal and the envelope spectrum of the IMF1 component. In the initial stage of rotor degradation, two characteristic frequencies of *f* = 16 Hz and *f* = 32 Hz were clearly observed in the frequency domain signal; there were two characteristic frequencies of *f =* 14.5 Hz and *f* = 21.5 Hz in the signal envelope spectrum. Among them, *f* = 16 Hz and *f* = 32 Hz were considered to be the characteristic frequencies of the whole meshing of the roller and the screw after the split rotor was attracted to its second harmonic, which may have been caused by insufficient meshing between the split rotor and the screw due to process deformation, etc.; *f* = 14.5 Hz and *f* = 21.5 Hz may have originated from the modulation spectrum formed by the characteristic frequencies of the bearings in the rotor components and the characteristic frequencies of the rollers themselves.

**Figure 10:**
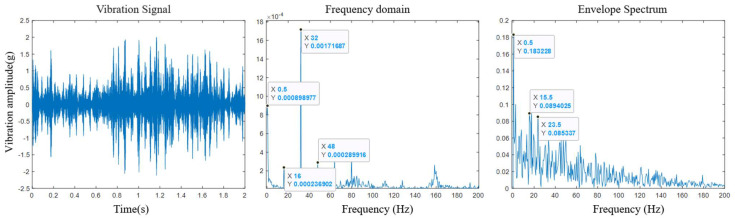
Demodulation results in the middle stage of the experiment.

As shown in Figure 10, by comparing the spectrum analysis results of the initial stage of the rotor component test with those of the initial stage of the comparative test, it was known that, after the rotor reached a certain stroke, the rollers rigidly connected to the screw rod were continuously worn. At this time, the second harmonic of the characteristic frequency of the overall component composed of the rotor and the screw rod significantly increased the energy component at the meshing frequency of the envelope spectrum, and the energy of the characteristic frequency of *f* = 32 Hz in the frequency domain signal was obviously enhanced. According to the previous analysis, the harmonics of this frequency originated from the insufficient meshing between the rollers and the screw rod. Therefore, the harmonic changes and modulation phenomena of the characteristic frequency can, to a certain extent, reflect the degradation process of the rollers, indicating that the wear of the rollers caused by the rigid connection changed their RUL. At the same time, by observing the envelope diagram, it was found that modulation spectra such as *f* = 15.5 Hz and *f* = 23.5 Hz appeared around the characteristic spectrum. The envelope signal showed certain modulation phenomena with the change in the rotor stroke, while the components of the frequency domain signal basically remained unchanged and only the amplitudes of some characteristics changed. It was seen that, when processing the data, a prediction model could be constructed according to the changes in the time-frequency domain characteristics.

**Figure 11:**
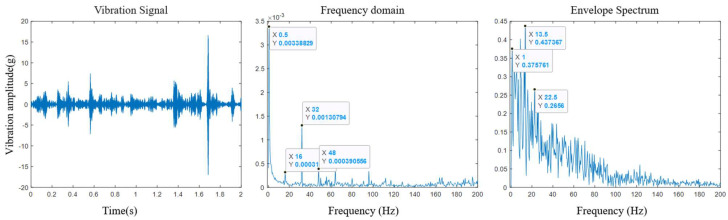
Demodulation results in the later stage of the experiment.

As the rotor stroke increased, the CRDM continued to degrade. As can be seen from Figure 11, the second harmonic of the characteristic frequency of the overall component composed of the rotor and the screw rod, *f* = 32 Hz, had a reduced energy at the meshing frequency in the envelope spectrum compared with the initial stage of degradation but a significant modulation phenomenon appeared near its envelope spectrum. In addition, when the rotor component and the roller component operated in meshing, unprecedented frequency bands appeared at the front and rear of the meshing frequency. This means that the changes in the characteristic frequencies of other components, or the degradation of the rotor itself, led to changes in the connection and transmission process between the components.

Based on the above analysis, it was inferred that the wear degree of the roller component was closely related to the energy characteristics around specific frequency points in the envelope spectrum.

#### 3.2.2. Feature Extraction of CRDM Roller Degradation

**Figure 12:**
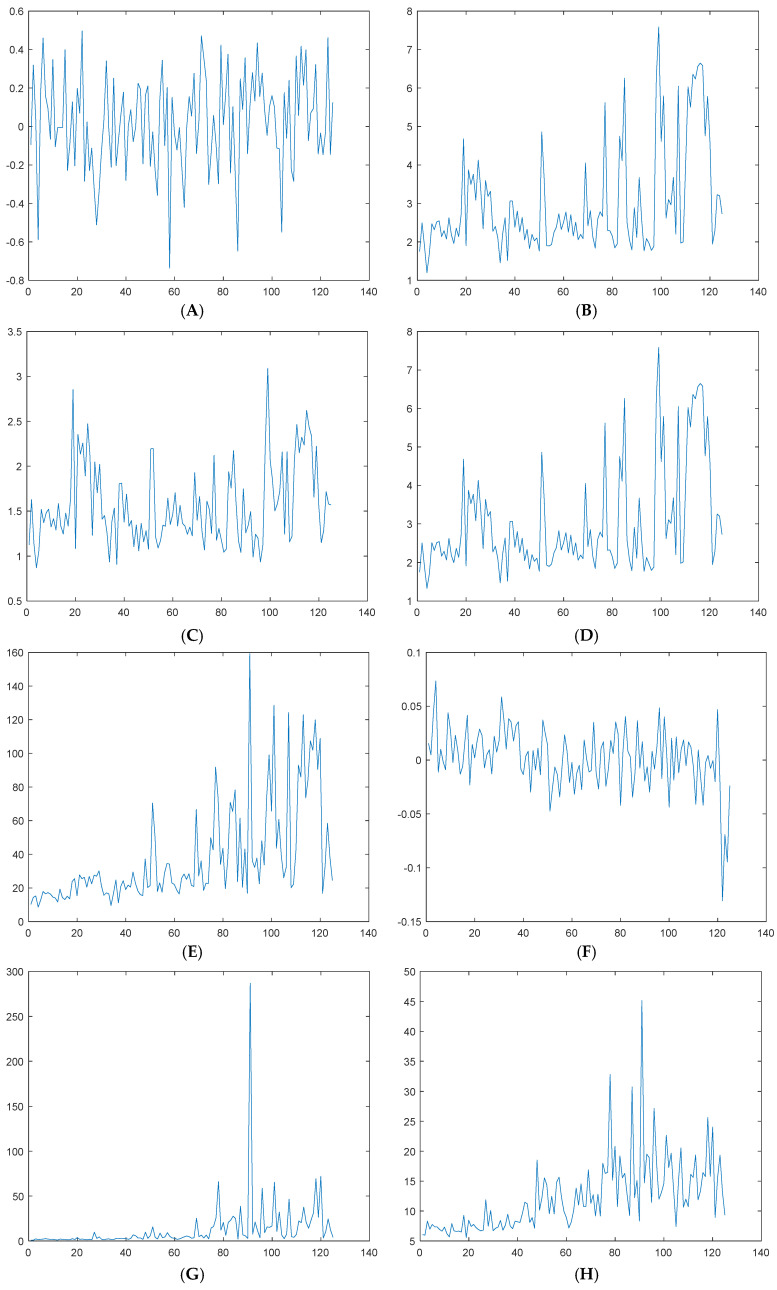

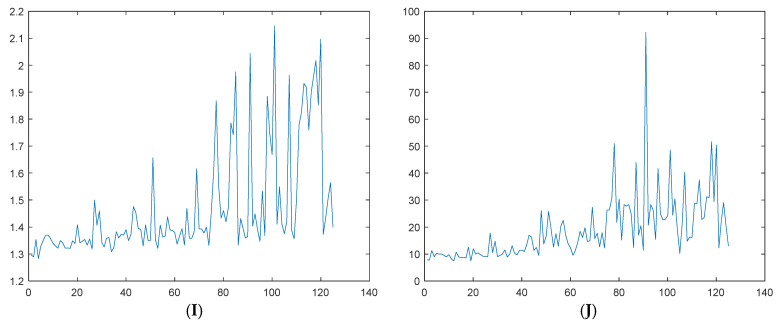
Time-domain characteristics: (**A**) mean value; (**B**) standard deviation; (**C**) square root amplitude; (**D**) RMS; (**E**) peak-to-peak; (**F**) skewness; (**G**) kurtosis; (**H**) crest factor; (**I**) waveform factor; (**J**) pulse factor.

We calculated the mean value, standard deviation, root mean square amplitude, root mean square, peak-to-peak value, skewness, kurtosis, peak factor, margin factor, form factor, impulse index, etc. of each segment of the vibration signal in the time domain. The obtained results are shown in Figure 12; the vertical axis in the figure represents the characteristic parameters and the horizontal axis represents the number of data groups.

**Figure 13:**
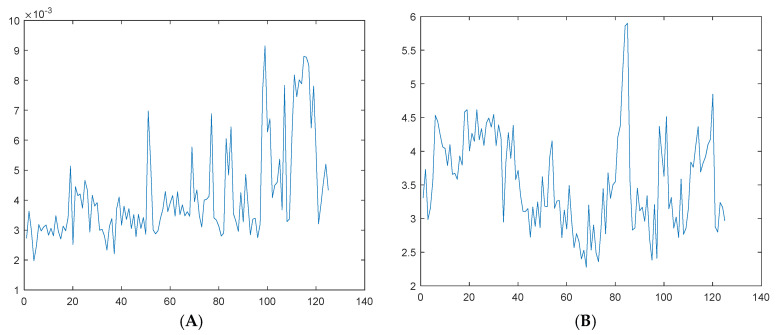
Frequency-domain characteristics: (**A**) mean frequency; (**B**) standard deviation of frequencies.

In the frequency domain, we calculated the mean frequency and standard deviation frequency of the selected frequencies according to the demodulation results of the segmented vibration data. The obtained results are shown in Figure 13.

#### 3.2.3. Analysis of the Degradation Trend of CRDM Rollers

In practical observations, we noticed that there was a significant correlation between the changing trends of some characteristic parameters and the wear state of the rotor components. These characteristic parameters exhibited obvious increasing or decreasing trends during the wear process of the rotor components, which could effectively reflect their wear situation. However, it should be particularly noted that not all characteristic parameters showed such a monotonic change pattern. Therefore, when screening the key characteristic parameters, multiple dimensions need to be comprehensively considered, including but not limited to the correlation between the parameters and the wear state, the stability of parameter changes, and the ease of obtaining the parameters.

After strict screening and comparison work, we finally determined five key characteristic parameters that can characterize the degradation trend of the rotor components. These parameters not only have high sensitivity and accuracy but also can comprehensively reflect the wear state of the rotor components. Specifically, as shown in Table 1, these parameters cover multiple key aspects of the rotor components, including dimensional changes, surface topography, and material properties, etc. The selected parameters can basically describe the degradation of various internal components of the rotor, such as rollers, large and small bearings, cages, etc. During the process of selecting signals, we tried our best to choose signals with monotonicity, which is beneficial for the learning of subsequent models.

Table 1 lists in detail the specific values of these key characteristic parameters and their changing trends. Among them, the standard deviation of frequency (A1) reflects the dispersion of the engagement frequency (e.g., 16 Hz and 32 Hz in Figure 8), which increases with the deviation of the roller screw due to wear. Skewness (A4) reflects sensitivity to shock signals caused by asymmetric wear (e.g., envelope spectral modulation in Figure 10). Standard deviation (A3) reflects the surface roughness of the roller component. As wear increases, the surface roughness gradually increases, which in turn adversely affects the friction performance and service life of the roller component. Impulse factor (A5) captures high-frequency impacts from surface defects (Figure 4). In addition, by comparing the changing trends among different parameters, we can conduct a more in-depth analysis of the wear patterns of the rotor components. For instance, the changing trends of certain parameters exhibit certain periodic characteristics, which may be related to the periodic loads on the roller component during its operation. Meanwhile, the correlations among different parameters also provide us with more information about the wear mechanism of the roller component.

In conclusion, through in-depth research and analysis of the key characteristic parameters of the roller component, we can more accurately grasp its wear situation during operation. This not only helps us formulate more scientific and reasonable maintenance and servicing measures to extend the service life of the roller component but also provides strong support for the optimized design and performance improvement of the CRDM system.

### 3.3. Operation Status Prediction of CRDM Rotor Components Based on BiLSTM

The prediction principle of the CRDM rotor is based on data analysis to predict its operating status and ensure the stable operation of the reactor. However, the complexity and nonlinear characteristics of the rotor vibration signals make it difficult for traditional models such as linear regression and time series analysis to accurately describe its motion laws. To overcome this challenge, for this paper we selected the BiLSTM method, which has become a promising prediction method due to its unique structure and powerful learning ability. At the same time, in order to compare the effects of different neural networks on the prediction of the rotor operating status, this work used multiple methods for comparison.

BiLSTM combines the advantages of LSTM and bidirectional RNN, taking into account the information before and after the input sequence and accurately capturing the sequential dependencies. In the prediction of the CRDM rotor, BiLSTM constructs an accurate model by learning the inherent laws of historical data. This model extracts useful feature information through encoding and decoding to predict the future state of the rotor. Compared with traditional models, BiLSTM has higher prediction accuracy and generalization ability. It optimizes its performance by learning a large amount of historical data, extracts useful information from complex behaviors, and accurately predicts future states. In addition, combining it with other algorithms can form more complex prediction models, improving stability and accuracy. Through theoretical analysis and practical applications, it provides strong support for subsequent algorithm research and offers a reliable guarantee for the safe operation of nuclear reactors.

#### 3.3.1. Grouping of Rotor Degradation Feature Data

Based on the data analysis in the previous section and the changing trends of the characteristic parameters obtained therefrom, this work defined the vibration signals collected within the stroke interval as samples of the roller components in a normal operating state during work. In the actual operating conditions, there were certain differences between the wear and degradation process of the roller components and the process simulated on the test bench. Considering this situation and in order to be able to diagnose the early wear state of the roller components as early as possible and obtain more abundant early fault characteristics, this work classified the vibration signals obtained within the stroke interval as sample data of the early fault state of the roller components.

**Figure 14:**
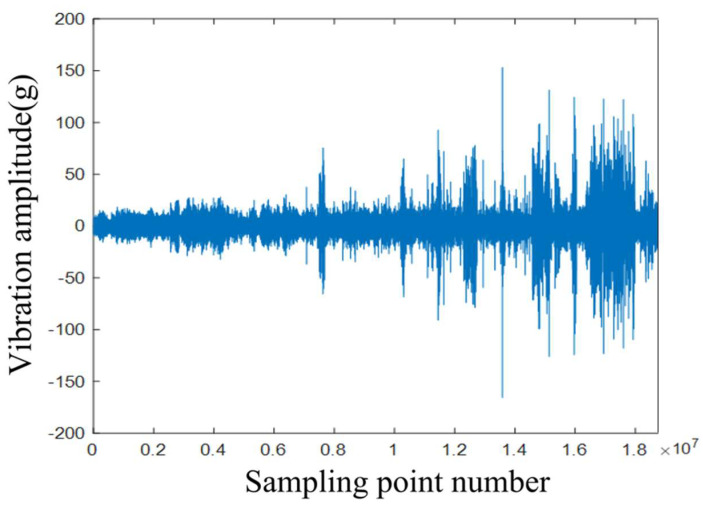
Full-life vibration data of the CRDM.

As shown in Figure 14, the data used in the rotor running state prediction in this paper were the full-life vibration data of the rotor of the control rod drive mechanism collected in the 3.1 life test, and the axial data in the three measurement signals were used. Currently, there are 125 groups of full-life vibration data collected. According to the above data grouping method, 100 groups were taken from the 125 groups of full-life vibration data as the training set and 25 groups were taken as the test set. The vibration data obtained for this paper came from the hot-state life assessment test, which simulates to a certain extent the working conditions of the drive mechanism in the actual environment. Therefore, the full-life characteristics of the early CRDM obtained in this paper could maintain a good consistency with the actual operation.

#### 3.3.2. Prediction Results of the Operating Status

The BiLSTM neural network used contained a sequence input layer, and the input was five degradation sensitive parameters (A1–A5, including frequency standard deviation, average frequency, standard deviation, skewness, and pulse factor), as defined in Table 1. After the input of the sequence, it entered the BiLSTM layer with four hidden units. The activation function used in the network was ReLU. After the output from the hidden layer, the full connection layer was entered, and then the regression output was carried out. The output was the CRDM remaining useful life label.

**Figure 15:**
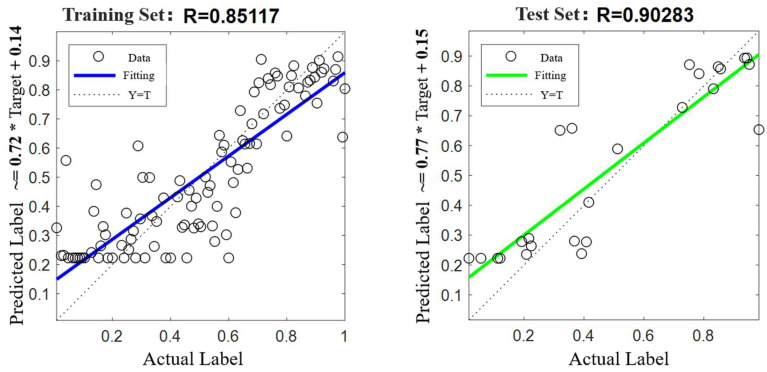
Prediction results of the operating status.

The time-frequency and features extracted from the raw vibration signals with the lifetime labels were directly fed into the model for training. The optimal hyperparameters were determined using a grid search method, using the “step” strategy in Pytorch as the learning rate annealing method, with an initial learning rate of 0.001 and decaying (multiplied by 0.1) at the 50th and 75th iteration cycles, respectively, with a batch size of 32 and 4 hidden cells. Python was the language and the programming framework was Pytorch 2.0. The training results were obtained, as shown in Figure 15.

In addition, we directly set the RUL representing health status as the running time before the end of the CRDM life. In this model, the input was the joint feature after feature selection, and the output RUL label was represented by the degradation percentage of the CRDM. The label was normalized, which was equal to 0 at the beginning of the CRDM operation and recorded as 1 when it ran to failure.

The remaining useful life of CRDM predicted by the model is shown in Figure 15. The left image clearly shows the prediction label and fitting curve of the training set, and the right image is the prediction label and fitting curve of the test set.

#### 3.3.3. Evaluation of the Prediction Effect of the Operating Status

In order to verify the superiority of the method adopted for this paper, a total of nine neural network regression prediction models, namely, BiLSTM, CNN, BP, Elman, RBF, Random Forest, LSBoost, SVR, and Adaboost, were selected. Using the five time-frequency domain features selected previously as inputs and the degradation features of the rotor of the CRDM as labels, supervised learning was carried out. Table 2 shows the comparison of R2, MAE, and RMSE of the training set and the test set of each model.

Loss functions are commonly used as indicators to judge the prediction errors of models, such as the mean square loss in regression prediction. However, in addition to this, using loss functions as evaluation indicators has some limitations and is not intuitive (for example, in classification tasks, f1-score is also commonly used, which can directly show the correct classification situations of various categories). Here, we mainly interpreted the commonly used error evaluation indicators for regression and classification prediction tasks, respectively. To evaluate the errors of regression models, a relatively simple idea is to “take the positive value” of the differences between the true values and the predicted values and then calculate the average, as follows:

(15) $$MSE=\frac{1}{n}\sum_{i=1}^{n}{\left(y_{i}-{\hat{y}}_{i}\right)}^{2},\in [0,+\infty )$$

(16) $$RMSE=\sqrt{\frac{1}{n}\sum_{i=1}^{n}{\left(y_{i}-{\hat{y}}_{i}\right)}^{2},\in [0,+\infty )}$$

(17) $$MAE=\frac{1}{n}\sum_{i=1}^{n}\left|y_{i}-{\hat{y}}_{i}\right|,\in [0,+\infty )$$

(18) $$R^{2}=1-\frac{\sum_{i=1}^{n}{\left(y_{i}-{\hat{y}}_{i}\right)}^{2}}{\sum_{i=1}^{n}{\left(y_{i}-\bar{y}\right)}^{2}}\in [0,1]$$

By observing the prediction results, it was found that LSBoost and Adaboost showed very obvious training effects on the training set, but their effects on the test set were obviously insufficient and a significant over-fitting phenomenon was observed. Continuing to observe the prediction effects of other models, it was found that the R2 values of the CNN, RBF, and SVR models on the test set were significantly lower than those on the training set. This indicated that the training effects of these three models on the data were not good. Through comparison with other methods, it was found that the R2 value of the BiLSTM model on the test set was significantly higher than that of other methods, which proves that the method adopted in this paper is more superior in terms of prediction accuracy compared with other methods.

Compared to other models, the bidirectional layer of BiLSTM can sense both past and future information, which is crucial for capturing the wear and tear trend of CRDM. Unlike convolutional networks, LSTM cells can suppress gradient vanishing to learn long-term dependencies. While other models have limitations when dealing with the lifetime prediction problem, CNNs encounter difficulties when dealing with continuous features; they perform poorly in time series prediction. SVR is unable to model nonlinear degradation dynamics. At the same time, when we continued to observe the characteristic parameters of the remaining methods, we found that the BiLSTM model also performed better in terms of the MAE of the test set and the RMSE of the test set. Therefore, from the comparison of the R2, MAE, and RMSE results of the training set and the test set of each model, it was concluded that the BiLSTM model had certain superiority when predicting with the time-frequency domain features screened in this paper as the input.

## 4. Conclusions

This paper addresses the challenge of predicting the remaining useful life of rotor components in the CRDM of floating nuclear reactors by proposing a novel method integrating VMD and BiLSTM networks. Through bench experiments, full-life vibration signals of the CRDM were collected and decomposed via VMD to suppress noise and extract intrinsic mode components related to roller degradation. Time-frequency domain analysis identified five key features (e.g., standard deviation of frequencies, skewness) with strong correlation to degradation trends. The developed BiLSTM model, trained on these features, achieved high prediction accuracy. By accurately predicting RUL, it supports safe and stable operation of floating nuclear reactors, reducing the risk of control rod failures due to wear. This research provides a new solution for predicting the operational status of CRDMs in floating nuclear reactors, which is important for the safe operation of nuclear reactors.

Although this research achieved certain results, there is still more work to be performed in the future. Specifically, it can further explore the mechanism of the influence of the dynamic load characteristics of the floating nuclear reactor on the equipment life in the simulated marine complex environment. At the same time, the CRDM life prediction method based on multi-channel data fusion is studied in depth, which provides more comprehensive theoretical support and technical reserves for the safe and reliable operation of the floating nuclear reactor.

## Figures and Tables

**Table 1:** Characteristic parameters for describing the degradation trend of rotor components.

Parameter Number	Parameter Name	Parameter Definition
A1	Standard Deviation of Frequencies	The discrete characteristic of frequency
A2	Mean Frequency	The centroid position of the signal energy
A3	Standard Deviation	The degree of dispersion of the signal distribution
A4	Skewness	The degree of deviation of the signal
A5	Pulse Factor	The instantaneous high-frequency components in the signal

**Table 2:** Comparison of parameters of each model.

Method	Training Set *R*^2^	Test Set *R*^2^	Training Set MAE	Test Set MAE	Training Set RMSE	Test Set RMSE
BiLSTM	0.7244	0.8151	0.11321	0.10546	0.14733	0.14151
CNN	0.88395	0.60579	0.072405	0.15613	0.095604	0.20013
BP	0.74495	0.74415	0.10542	0.11738	0.14173	0.16123
Elman	0.75247	0.77462	0.10886	0.11	0.13963	0.15132
RBF	0.7872	0.59382	0.99053	0.14544	0.12946	0.20314
RF	0.87332	0.71843	0.074756	0.13407	0.099886	0.16914
LSBoost	0.99948	0.75265	0.0031423	0.12401	0.0062957	0.15145
SVR	0.80767	0.66059	0.0084103	0.1417	0.12308	0.1857
Adaboost	1	0.31665	0.0005511	0.18416	0.0002756	0.25173

## Data Availability

The datasets presented in this article are not readily available because the data are part of an ongoing study.
